# 7.4 PHENOTYPIC AND GENETIC ASSOCIATIONS BETWEEN SCHIZOPHRENIA AND RETINAL VESSEL DIAMETER

**DOI:** 10.1093/schbul/sby014.026

**Published:** 2018-04-01

**Authors:** Madeline Meier, Enda Byrne, Nicholas Martin, Tien Wong, Xueling Sim

**Affiliations:** 1 Arizona State University; 2 University of Queensland; 3 Queensland Institute for Medical Research; 4 Singapore Eye Research Institute; 5 University of Michigan

## Abstract

**Background:**

Individuals with schizophrenia are at increased risk for cardiovascular diseases, and this risk cannot be fully explained by antipsychotic medications or lifestyle factors. Retinal imaging offers a non-invasive means of visualizing the microvasculature in living individuals with schizophrenia. Here we test whether individuals with schizophrenia exhibit retinal microvascular abnormality (Meier et al., AJP, 2013), and test for overlap in the genetic variants associated with schizophrenia and retinal microvascular abnormality.

**Methods:**

To test whether individuals diagnosed with schizophrenia showed microvascular abnormality, we used data from the Dunedin Study, a representative cohort of 1,000 New Zealanders followed from birth to age 38. The cohort underwent retinal imaging at age 38, and retinal venular (small veins) and arteriolar (small arteries) diameters were obtained. Analyses compared individuals with schizophrenia (n=27), healthy individuals (n=412), and individuals with medical or psychiatric conditions (ns ranged from 110–210) on retinal vessel diameter. To test for overlap in the genetic variants associated with schizophrenia and retinal vessel diameter, we used linkage disequilibrium-score regression (LD regression) to obtain the genetic correlation between schizophrenia and retinal vessel diameter. This method requires only GWAS (genome wide associate studies) summary statistics rather than actual genotypes. Summary statistics for schizophrenia and retinal vessel diameter came from published meta-analyses.

**Results:**

Adults diagnosed with schizophrenia had wider retinal venules (standardized mean=0.59) compared with same-age healthy adults (standardized mean=-0.20) and compared with individuals diagnosed with hypertension, diabetes, and tobacco dependence. Findings could not be explained by antipsychotic medication, as venular diameter was similar in the subset of individuals diagnosed with schizophrenia who had not taken antipsychotic medication in the year prior to retinal imaging (n=22; standardized mean=0.69). There were no differences in arteriolar diameter between individuals diagnosed with schizophrenia and all other groups. Results from LD regression showed a small genetic correlation between schizophrenia and venular diameter (r=0.05, p=.31) and a slightly larger genetic correlation between schizophrenia and arteriolar diameter (r=0.17, p=.02).

**Discussion:**

Wider venular diameter is a distinguishing feature of schizophrenia, but genetic variants associated with schizophrenia overlap more strongly with variants associated with arteriolar diameter. It is possible that environmental influences associated with schizophrenia tend to narrow arterioles, obscuring a phenotypic link between schizophrenia and wider arterioles. Pathophysiological mechanisms underlying vessel diameter, including inflammation and endothelial dysfunction, might be related to the development of schizophrenia, and particular genes might contribute to both schizophrenia and arteriolar diameter. Findings will be discussed in relation to links between retinal vessel diameter and IQ (Shalev, Meier et al., Psychol Sci, 2013) and depression (Meier et al., Psychosom Med, 2014).

